# Supplementary material for: Role of syn-eruptive plagioclase disequilibrium crystallization in basaltic magma ascent dynamics
Source: Nat Commun. 2016 Dec 12;7:13402. doi: 10.1038/ncomms13402 (PMC5159818; doi:10.1038/ncomms13402)
Supplement: Supplementary Information — Supplementary Figure 1, Supplementary Note 1 and Supplementary References. [file ncomms13402-s1.pdf]

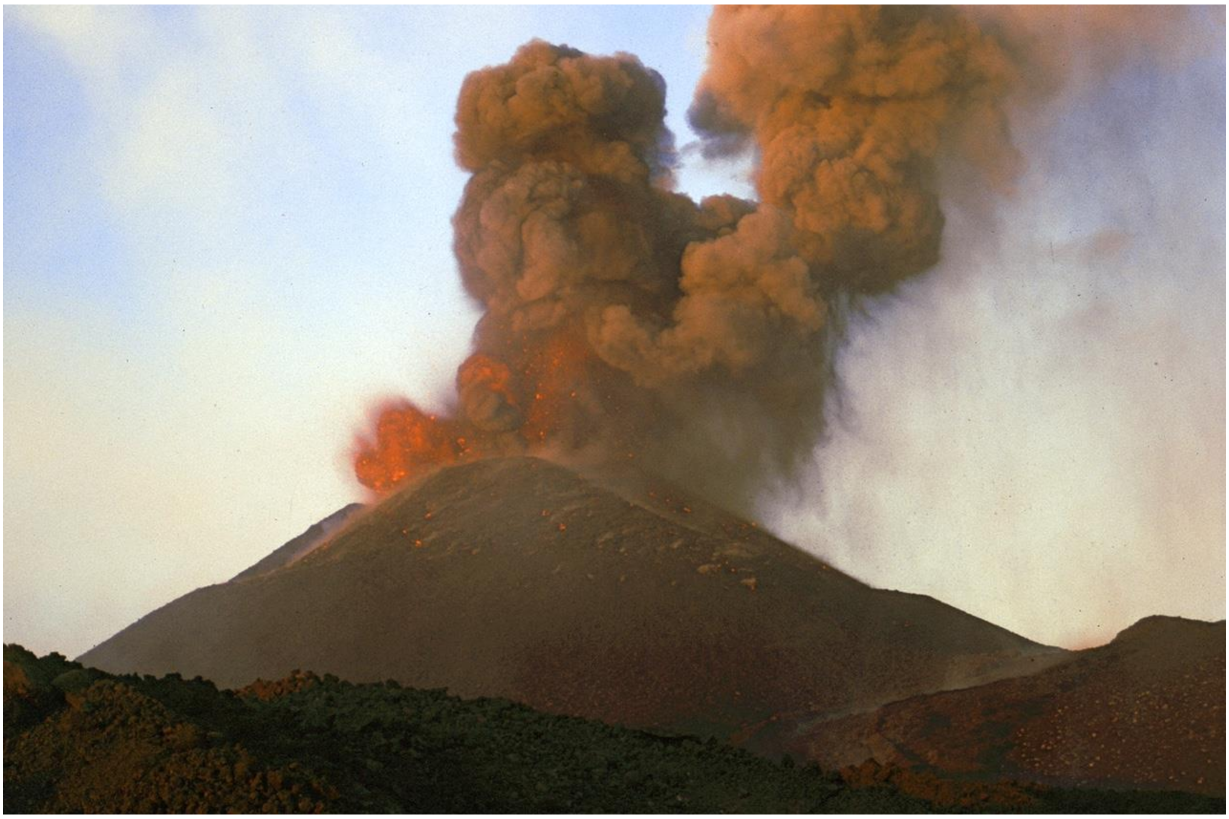

Supplementary Figure 1: Etna 2001 activity

Eruptive activity at the 2550 m vent during the 2001 flank eruption at Mount Etna (photograph courtesy of Dr. Boris Behncke, INGV Osservatorio Etneo).

Supplementary Note 1

The 2001 flank eruption is one of the most studied eruptive episodes at Mt. Etna[1-11]. This eruption started at 17 July and ended at 9 August and was active from seven fissures at different altitude, showing different eruptive styles: fire fountains, Strombolian activities and lava effusions. Here we give a brief description of this event.

During the night of 16-17 July, a paroxysmal event occurred at the South East Crater (SEC) from a first fissure (F1) at 3080 m a.s.l.. This eruptive episode was followed by a lava outflow from a second fissure (F2) located at 2950 m a.s.l., which continued at a low effusion rate for a few days after the paroxysm. A couple of hours after the paroxysm, the third fracture (F3) opened between 2690 m and 2895 m a.s.l., producing a copious lava flow. On 18 July the fourth fissure (F4) at 2100 m a.s.l. became active, producing a lava flow that extended southward invading the parking area between the Rifugio Sapienza and the Monti Silvestri and continuing its advance towards the town of Nicolosi. On 19 July lava fountaining was observed from this fracture, while in the afternoon of the same day a new cluster of vents (F5) opened at 2550 m a.s.l., producing violent explosions, and the formation of dense ash and lapilli column rising 200-300 m high. On 20 July a sixth new fracture system (F6) at 2600 m a.s.l. became active showing a Strombolian activity and a small lava flow. On the same day and on the following ones, strong lava fountaining and phreatomagmatic activities were observed respectively from F4 and F5 (Supplementary Fig. 1). On 23 July a small lava outflow was emitted from a new fissure (F7) at 3060 m a.s.l.. After a temporary decrease in the eruptive activity on 24 July, a change from phreatomagmatic to magmatic activity was observed on 25 July. Effusive activity began from F5 on 26 July and simultaneously the effusion rate at F4 showed a decrease. On 26 July the activity at F1 and F7 ceased and a decrease of effusion rate in the other still active fissures was observed in the following days. On 29 July the activity from F6 came to an end, while on 30 July a new major lava flow was erupted from F5. On 1 August the activity at F5 shifted back to phreatomagmatic and almost simultaneously the activity at F2 ended. The remaining active vents in the following week were F3, F4 and F5, but the intensity of the explosions diminished progressively, completely ending on 9 August. The location

of the eruptive vents of the 2001 flank eruption at Mt. Etna, and the lava flow fields produced during the eruption are indicated in the comparative maps illustrated in ref. 3.

The total volume of all lava emitted during the eruption[3,6] was about 25 x 10^6 m^3 (dense rock equivalent, DRE) while the tephra volume was about 5-10 x 10^6 m^3. The estimation of mean volume flow rate during the whole eruption was about 11 m^3/s with a peak of 31 m^3/s on 22 July[12]. The petrography analysis of the erupted scoria showed that there were two different types of product: one with a large crystal content (from 30 to 39 vol.%) and another one with a small content (from 15 to 23 vol.%). The scoria with small crystal content was erupted from vents below 2600 m a.s.l. (i.e. F4 and F5, hereafter Lower Vents, LV), while the other was erupted from vents above 2600 m a.s.l. (i.e. F1, F2, F3, F6 and F7, hereafter Upper Vents, UV). Another important difference between these two scoria is the plagioclase content. In the LV products the plagioclase content is 4-7 vol.%, while in the UV scoria is 16-23 vol.%[1]. These differences suggest that the 2001 flank eruption was supplied by two different types of magma, one nearly identical to the magma emitted at Etna during the last few decades (UV magma), and another one that ascended vertically from a different reservoir (LV magma)[1,3,4].

Supplementary References

1 Corsaro, R. A., Miraglia, L. & Pompilio, M. Petrologic evidence of a complex plumbing system feeding the July-August 2001 eruption of Mt. Etna, Sicily, Italy. *B. Volcanol.* **69**, 401-421 (2007).

2 Métrich, N., Allard, P., Spilliaert, N., Andronico, D. & Burton, M. 2001 flank eruption of the alkali- and volatile-rich primitive basalt responsible for Mount Etna's evolution in the last three decades. *Earth Planet. Sc. Lett.* **228**, 1-17 (2004).

3 Behncke, B. & Neri, M. The July-August 2001 eruption of Mt. Etna (Sicily). *B. Volcanol.* **65**, 461-476 (2003).

4 Lanzafame, G. et al. Structural features of the July-August 2001 Mount Etna eruption: evidence for a complex magma supply system. *J. Geol. Soc. London* **160**, 531-544 (2003).

5 Calvari, S. Multidisciplinary approach yields insight into Mt. Etna 2001 eruption. *Eos Trans. AGU*, **82**(52), 653-656 (2001).

6 Coltelli, M., Del Carlo, P. & Scollo, S. Physical parameters of the ash fallout occurred during the 2001

eruption of Etna and implication for volcanic hazard assessment (Abstract). In: *Proc. Assemblea 1 Anno*, 241-242 (2001).

7 Lautze, N. C. et al. Pulsed lava effusion at Mount Etna during 2001. *J. Volcanol. Geoth. Res.* **137**, 231-246 (2004).

8 Taddeucci, J., Pompilio, M. & Scarlato, P. Conduit processes during the July-August 2001 explosive activity of Mt. Etna (Italy): inferences from glass chemistry and crystal size distribution of ash particles. *J. Volcanol. Geoth. Res.* **137**, 33-54 (2005).

9 Viccaro, M., Ferlito, C., Cortesogno, L., Cristofolini, R. & Gaggero, L. Magma mixing during the 2001 event at Mount Etna (Italy): effects on the eruptive dynamics. *J. Volcanol. Geoth. Res.* **149**, 139-159 (2006).

10 Bonforte, A., Gambino, S. & Neri, M. Intrusion of eccentric dikes: the case of the 2001 eruption and its role in the dynamics of Mt. Etna volcano. *Tectonophysics* **471**, 78-86 (2009).

11 Favalli, M., Harris, A. J., Fornaciai, A., Pareschi, M. T. & Mazzarini, F. The distal segment of Etna's 2001 basaltic lava flow. *B. Volcanol.* **72**, 119-127 (2010).

12 Coltelli, M. et al. Analysis of the 2001 lava flow eruption of Mt. Etna from three-dimensional mapping. *J. Geophys. Res.: Earth Surf. (2003–2012)* **112**(F2) (2007).
